# Would a simple attention-reminder in discrete choice experiments affect heuristics, preferences, and willingness to pay for livestock market facilities?

**DOI:** 10.1371/journal.pone.0270917

**Published:** 2022-07-08

**Authors:** Girma T. Kassie, Fresenbet Zeleke, Mulugeta Yitayih Birhanu, Riccardo Scarpa

**Affiliations:** 1 Social, Economics, and Policy Research Team, International Center for Agricultural Research in the Dry Areas (ICARDA), Addis Ababa, Ethiopia; 2 School of Agricultural Economics & Agribusiness, Haramaya University, Dire Dawa, Ethiopia; 3 Livestock Genetics, International Livestock Research Institute (ILRI), Addis Ababa, Ethiopia; 4 Business School, Durham University, Durham, United Kingdom; Groupe ESC Dijon Bourgogne, FRANCE

## Abstract

This study addresses the question whether an ‘attention reminder’ in discrete choice experiments (DCE) affects preferences, willingness to pay (WTP), and attribute non-attendance (ANA). We report on an experiment which elicited preferences for livestock market facilities from 960 randomly selected farm households in Ethiopia. Basic diagnostic comparisons of the estimations showed that taste parameters are significantly different and the WTP values of two (out of eight) facilities are different between *before* and *after* the reminder. Latent class model based ANA analysis revealed that the reminder has increased fully compensatory choice behavior [full attention] among sample respondents. The mixed logit models estimated in WTP space also showed that the WTP values are slightly smaller for most of the facilities after the reminder. In terms of relative importance, veterinary clinic, fenced shed, and watering trough facilities are the three livestock market facilities valued most by the farm households both *before* and *after* the reminder. Our results imply that researchers studying behaviors of rural communities in developing countries using DCEs might be able to address issues related to heuristics if they reminded respondents of the need to pay attention to all elements in the experiment unless understanding the choice decision making process itself is the point of interest. Empirically, livestock market development initiatives need to take into account farmers’ clear and consistent prioritization of the market facilities.

## 1. Introduction

The success of rural development initiatives depends on the importance of the social constraints they are meant to address. The importance of the challenges faced by the society in turn depends on the experiences and intentions of each member of the society. Therefore, interventions that aim at improving livelihoods of a given society are expected to emanate from the choices the individuals make in relation to the components or the entire intervention. Economic choices of an individual are manifestations of his/her preferences given the circumstances. The differences in experiences, intentions, and constraints among individuals make the science of group level preference elicitation and choice analysis an arduous task.

This task becomes more complicated when the commodity in question is new or when it is a hypothetical combination of observed attributes. We intend to elicit preferences and willingness to pay for livestock market facilities in Ethiopia. There are no any facilities in the livestock markets in Ethiopia and yet the facilities are known to the rural communities [1,2]. It can be assumed that the rural communities would be generally interested in the market facilities. However, the heterogeneity we implied above manifests itself in the choice behavior of the individuals in the community rendering aggregate descriptions less reliable. Using discrete choice experiments, we intend to see whether choices of market behavior are consistent *before* and *after* a reminder that aims at encouraging respondents to consider all facilities in the hypothetical market facilities.

Discrete choice experiments [DCEs] are multi-attribute based instruments for eliciting stated preferences and for estimating willingness to pay for attributes of quality-differentiated goods and services [3–5]. DCEs are supposed to be close-to-reality representation of what the respondents face in the real markets of goods and services. When carefully designed and implemented, DCEs have proved to be very useful and relevant tools to analyze preferences and, hence, willingness to pay for the different attributes that characterize the commodity [6].

Theoretically, Lancaster’s characteristics theory of value [7] and McFadden’s random utility theory [8] form the basis of DCEs. There are other underlying assumptions made in estimating the perceived relative utility that supposedly drives the choice decisions. The application of DCEs assumes compensatory decision-making and well-formed preferences. Compensatory choice implies that respondents consider all or most of the available information and make trade-offs between product attributes [9]. Well-formed preferences imply that individuals have consistent preferences and they can retrieve an appropriate response to any preference-elicitation question [10,11].

These assumptions imply that the consumer is expected to be a rational decision maker, portraying behavior as a planned and consistent activity, which aims to maximize some subjective measure of value [11]. Rationality is, however, bounded because of the limitations the decision makers have in thinking capacity, available information, and time [12]. Economists and psychologists have reported detailed accounts of consumer behavior that do not necessarily synchronize with the neo-classical theories of ‘rational’ consumer and ‘consistency’ in choices. Non-compensatory and adaptive decision-making have been observed among consumers in all lifestyles [13–18].

When faced with complex choice decisions, individuals employ cognitive shortcuts or heuristics to simplify the choice decisions under uncertainty [4,19]. Heuristics in DCEs, include attribute non-attendance, anchoring, imposing thresholds on attribute levels to represent acceptable levels, and attribute aggregation where they are in common units [9,20].

The need to understand the mechanics with which respondents are making decisions in DCEs is growing in importance along with the popularity of DCEs as preference elicitation tools. DCEs need to embed tests for response distortions that are commonly observed in cognitive experiments, such as anchoring to cues in the elicitation format, reference point or status quo bias, extension neglect, hypersensitivity to context, and shadowing from earlier questions and elicitations [3].

Empirical evidence shows that pooling observations where some respondents attend to all attributes while others attend to only a subset would lead to erroneous and biased estimates [21]. Similarly, [22] indicated that taking into account heuristics—in the form of attribute non-attendance—improves model fit with little or no effect on marginal rates of substitution. The predominant recommendation is, however, that including the decision process in the analytical models is necessary and informative given the context [21,23–25].

Quite a lot has been done in identifying the types of heuristics in choice experiments, their causes, and their impact on preferences and willingness to pay for attributes or attribute levels of the hypothetical profiles (see [4] and [26] for a detailed discussion). There is, however, a general consensus that there is very little of the decision processing literature incorporated into discrete choice modeling which is increasingly becoming the mainstream empirical context for preference measurement and willingness to pay derivatives [4]. There is a focus on the outcomes in analyzing economic decisions [27]. We agree with [10] that no studies have so far made detailed investigations of decision making in conjoint experiments and how elements of different decision strategies are combined in order to make choices.

In this study, we looked into the effects of a simple ‘*attention reminder’* on key parameters of demand functions estimated based on livestock market DCEs. Behavioral economists argue that a *nudge* in the form of a reminder can have a significant impact on how decision makers behave [28,29]. A nudge is any aspect of the choice architecture that alters people’s behavior in a predictable way without forbidding any options or significantly changing their economic incentives [29]. We consider the simple attention reminder more of as an information nudge than a cheap talk. Cheap talk focuses on minimizing hypothetical bias in stated choice methods and usually uses a much longer text to describe what the respondents shall be doing in their decision making process. In our case, we just reminded them and did not imply in any way that they shall pay attention to all facilities nor did we indicate any purpose the reminder is to serve.

To count as a mere nudge, the intervention must be easy and cheap to avoid. Our attention reminder can be considered as a simple nudge and it was framed for all respondents as “***Some of the respondents I talked to before you were not paying attention to all of the services while comparing the markets*. *We expect respondents to consider all the services in comparing the two markets on each of the choice cards***.” Our nudge references peer activity and is expected to increase cognition and get people think a little bit more about their decision-making process. The reminder nudge is expected to influence the choice behavior of our respondents without compulsion to act on it.

To analyze this causality we estimated conditional logit, latent class, and mixed logit models. This study therefore addresses the following three questions; (i) does a reminder nudge create a significant difference in taste parameters and WTP values (Sections 3.2 and 3.5)? (ii) Would a reminder nudge affect the extent and source of preference heterogeneity (Section 3.3)? And (iii) would a reminder nudge affect the choice simplification strategies of respondents (Section 3.4)?

The findings of the paper contribute in two important ways. First, Ethiopia is formulating policies that intend to the livestock sector including livestock market developments. There is very little information on the preferences of livestock market facilities that can be useful for macroeconomic decision-making. This research is based on a nationally representative data and, hence, it will inform the current policy formulation process. Secondly, we address an important theoretical and empirical question on whether a simple information/attention reminder can influence the heuristics decision makers adopt in DCEs. Our observation is that the nudge increases fully compensatory choice behavior, and given the fact that local communities never had a say in policy design and objectives and their lack of experience with market facilities, nudging should provide a more comprehensive mapping of the preferences which will certainly help designing market development plans.

## 2. Methodology

### 2.1. Research context

The role markets play in the livelihoods of rural communities in a developing country context can hardly be overemphasized. Ethiopia is a country with an agrarian economy where agriculture accounts for about 50% of the gross domestic product, employs 85% of the national labor force, and generates 90% of the foreign earnings. Ethiopia claims to have the largest livestock population in Africa and yet the sector contributes only about 27% of the agricultural value added [30,31]. Despite the relatively large livestock population, the country’s economy struggles with the low and declining performance of the sector. One of the critical challenges the livestock sector faces is a lack of efficiency of its marketing system. Livestock markets are usually marginal and/or abandoned plots of land with little or no facility. If there is any facility, it is usually a fence around the market mainly for fee collection and sheds for collectors. Otherwise, lack of infrastructure, limited physical accessibility, and bargaining power skewed towards the traders characterize rural livestock markets in the country [2].

Cognizant of the indispensable role of the markets in the growth and transformation agenda of the country, the government of Ethiopia has integrated market improvement in its recently developed livestock master plan and livestock sector analysis [32,33]. These plans are developed with considerable level of uncertainty due to lack of grass roots level information on the interest in and willingness to pay for market development interventions. We are, therefore, estimating the demand for the key market facilities that rural communities would like to have in the markets they depend on.

Lack of market services significantly undermines the market-based revenue margins livestock keepers generate from their production and elevate their cost of agricultural inputs. This limits the financial sustainability of the supply chain and impedes forward planning in herd composition, making the overall herd management unsustainable or suboptimal from the resilience viewpoint in the long run. Transaction costs of agricultural markets in general are quite high and choosing the livestock market to go to is one of the most salient decisions farmers make. In rural livestock markets, such costs are particularly high due to, among others, lack of transport facilities that force marketers to trek their animals, exposing herds to lack of feed and watering services on their way to and around markets, poor access to veterinary services, and lack of other handling facilities.

Establishing and sustaining these facilities in or around the livestock markets needs to be an integral part of the plans developed. In our context of study, markets are physically owned by the government–as it is the sole owner of land in Ethiopia–and yet it is the marketers’ demand for the market facilities that determines to what extent these facilities would help the rural communities make more out of their livestock. It is, therefore, imperative for self-sustainable infrastructure investment, to emphasize the need for understanding the willingness to pay for access to marketing services and their potential impact on the marketing performance of smallholder livestock keepers.

### 2.2. Sampling

This study assesses the preferences and WTP for market services identified by farmers and traders in three sites in different parts of the country. The sites are *Abergelle* in Northern Ethiopia, *Menz* in central north Ethiopia, and *Horro Gudru* in central west of Ethiopia. *Menz* represents the subalpine sheep dominated livestock production systems. *Horro Gudru* represents the highland crop-livestock mixed production systems, and *Abergelle* represents the goat dominated highland livestock production systems. The sites represent the three dominant livestock production systems in the highlands of Ethiopia where most of the country’s livestock wealth is found. In these sites, we covered seven administrative districts where livestock are crucial component of the rural livelihoods. Markets are places where farmers visit almost every week not only to buy and sell but also to have social interactions and garner information. Therefore, our population was the rural community in the selected sites. The list of residents was acquired from the administration offices in each of the districts. Then, we used systematic random sampling to select sample households. In Menz area, we randomly sampled 120 households from each of the three districts, namely, *Menz Gera*, *Menz Keya*, and *Menz Mamma*. In Horro Gudru, we randomly selected 240 farm households from *Horro* and 120 from *Jimma Geneti* district. In Abergelle site, we selected 120 farm households from each of the two districts, *Sekota* and *Abergelle*. The total sample size is, therefore, 960 farm households.

### 2.3. Choice experiment

We used a discrete choice experiment to elicit the preferences of survey respondents. A series of structured meetings with farmers, livestock traders, and local development agents enabled the identification of market shed with and without fences, veterinary clinic, resting or holding sheds, watering trough, toilet, and feed stalls or shops as the most important services livestock markets needed to have in the study sites (Table 1). The descriptions of the facilities included in the study and farmers key justifications in prioritizing the facilities are summarized as follows:

- *Sheds and fences*: The livestock markets in central Ethiopia are marginal plots of land in or close to the administrative capitals of Kebeles (Kebele [plural Kebeles] is the smallest unit of administration in Ethiopia) or districts. The animals and the marketers have no sheds to protect themselves from the scorching sun or the heavy rainfall. This creates a lot of pressure particularly on farmers as they will be rushed to avoid these inconveniences. In fact, in the rainy season, farmers avoid markets altogether, as there will hardly be any buyers in the market. Therefore, the availability of the sheds will be an important investment to increase farmers participation [visiting the markets anytime they want to] and staying as long as they think is right instead of being pushed away by the heat or the rainfall. This is, therefore, one of the services farmers identified as important and hence was included in the experiment.
- *Toilets*: This was an important service for women farmers in particular. They indicated that it is much easier to the male farmers to stay long in the market as the lack of toilet is not as stressful for men as it is for women. The discomfort that the lack of the service creates was mentioned as one of the reasons for women to be less interested to be in the livestock markets in the study areas. In fact, men farmers and traders indicated that the lack of toilets in or around the markets is an important challenge as there is no access to public toilet in these rural villages.
- *Watering troughs and feed shops*: Livestock are trekked to the market for hours usually without any feed and water supply along the road. Feed and water is not available for the animals in the markets either. The animals are usually exhausted and in many cases emaciated as the trips are frequent and long. The lack of water and feed forces farmers to sell their animals in prices less than they normally expect as trekking the animals back and forth further undermines the marketability of the animals. Therefore, farmers and traders were keen in having water troughs and feed stalls in or nearby the markets so that the animals remain well fed and marketable.
- *Veterinary clinics*: The farmers have no guarantee that the animals they are selling or buying are healthy. The buyers and sellers hardly meet again once the transaction is over. Given the risk of pests and diseases, therefore, the farmers can hardly afford to take any risk in buying an animal without clear medical background. So, they usually check for external symptoms and depend on the sellers’ words. The availability of the service will increase the bargaining power of the seller as he or she can easily verify the health status of the animals and, for the buyer, it increases the willingness to pay for the animal.
- *Holding sheds*: Farmers were so unhappy about the taxing system whereby tax is collected per animal every time it is brought to the market whether it is being transacted or not. Farmers indicated that they usually bring a herd of livestock together to easily trek the animals even if they are to sell one or two animals. They would like to have places where they can keep the animals not meant for selling and hence avoid paying taxes on them. Therefore, farmers strongly suggested holding barns to save some transaction cost of livestock marketing.

**Table 1:** Services (attributes) and delivery levels in the discrete choice experiment.

Service	Levels
Market shed	No shed Unfenced market shed (SUNF) Fenced market shed (SFEN)
Veterinary clinic close to the market	No Yes (VET)
Resting/holding shed close to the market	No Yes (HLD)
Watering trough in the market	No Yes (WAT)
Toilet in the market	No toilet Toilet with a cleaner (TCLN) Toilet with no cleaner (TNCL)
Feed stall/shop in the market	No Yes (FDSH)
Service charge/sold sheep	5 Eth Birr 7.5 Eth. Birr 10 Eth. Birr 12.5 Eth. Birr

The design of the choice experiment was articulated in two stages. First, we developed an efficient design with flat or no priors. Then, we collected data on 20 respondents in the study area, analyzed the data, generated priors and developed a Bayesian efficient design. The design had 48 profiles of markets facilities, described by different combinations of services and service levels. We blocked the profiles into two and added an opt out option in each of the choice sets. Each respondent was presented with 12 choice situations and asked to choose his/her preferred alternative. The reminder was introduced right after the sixth choice situation. The choice situations were presented in a random order both before and after the reminder. The opt out option was mentioned with the two alternatives in each of the choice sets.

The data were collected through paper questionnaire based in-person interviews. The interviews started with reading of consent statement and approval of the respondent to continue or otherwise. The consent statement included, *inter alia*, objectives of the study, sampling, confidentiality, anonymity, and request for confirmation of consent for participation. Only those who agree to participate were interviewed and even after consent respondents were clearly informed that they did not have to answer to questions should they feel so. In our sample, all sample households gave their oral consent to be interviewed.

### 2.4. Analytical framework

#### Taste parameters and WTP values

Choosing an alternative in a choice situation is an intricate behavioral decision where both the process and the outcome are important. Responses collected in DCEs are usually analyzed based on the foundations established by Lancaster’s characteristic theory of value [7] and McFadden’s random utility theory [8]. Therefore, sample respondent’s decision on which livestock market to visit is expected to be the result of marketers’ interest in the different facilities in the markets and the chosen market is expected to be the one that maximizes the perceived relative utility. Assuming that the individual (*n*) intends to maximize utility, the probability that an alternative market (*i*) in a given choice situation (*C*_*t*_) is chosen is equivalent to the probability that the perceived utility from alternative (*U*_*i*_) is higher than the perceived utility from the other alternatives (*U*_*j*_, *where j* = 1, *i*,…*j*, & *i* ≠ *j*) in the choice set. This can be formulated as:

$$P(i|C_{nt})=P(U_{nit}>U_{njt}),\;\forall i\neq j$$ (1)

We assume the utility function is linear in the explanatory variables and utility is separable in price, *p*, and the other facilities, *x*_*njt*_. Therefore, we can write the utility function as:

$$U_{nit}=-\alpha_{n}p_{njt}+\beta_{n}^{\prime }x_{njt}+\epsilon_{njt}$$ (2)

where *α*_*n*_ is the marginal utility of additional unit of market fee, and *β*_*n*_ is conformable vector of unknown individual-specific utility coefficients. *x*_*njt*_ is a vector of explanatory variables including facilities defining the alternatives and interactions of the facilities and *reminder dummy*, and *ϵ*_*njt*_ is Gumbel distributed with variance given by $\sigma_{n}^{2}\left(\frac{\Pi^{2}}{6}\right)$, where *σ*_*n*_ is an individual scale parameter. Dividing Eq 2 by *σ*_*n*_ does not affect behavior and results in a new error term which is independently and identically distributed extreme value type I with variance equal to Π^2^/6 [34,35]. The division results in:

$$U_{nit}=-{(\alpha }_{n}/\sigma_{n})p_{njt}+(\beta_{n}/\sigma_{n})\prime x_{njt}+\epsilon_{njt}/\sigma_{n}$$ (3)

The utility coefficients are therefore defined as *λ*_*n*_ = *α*_*n*_/*σ*_*n*_ and *c*_*n*_ = *β*_*n*_/*σ*_*n*_, while the error term becomes *ζ*_*njt*_ = *ϵ*_*njt*_/*σ*_*n*_. Using these derived coefficients, the utility function in preference space can be specified as:

$$U_{njt}={-\lambda }_{n}P_{njt}+c_{n}^{\prime }x_{njt}+\zeta_{njt}$$ (4)

We estimated the parameters of the utility function using mixed logit models. We report different versions of the mixed logit model estimated in both preference and WTP spaces. The estimations in preference space were used to derive WTP values, ratio of estimated coefficient of a facility to that of market fee, for the purpose of comparing them *before* and *after* the reminder. Poe test was conducted to look into the statistical significance of the differences between the WTP values [36]. Poe test applies complete combinatorial method to test for differences in the WTP values of two samples.

Comparison of heterogeneity in mean preferences was also done based on estimations of mixed logit in utility space. The variables considered as potential sources of heterogeneity were location, gender of respondent, age of respondent in years, household size, distance from market in walking hours, and small ruminant ownership in tropical livestock units.

Then, we estimated the utility functions in WTP space. Estimating the utility function in WTP space involves estimating the distribution of willingness to pay values directly by re-formulating the model in such a way that the coefficients represent the WTP measures [37].

We note that the WTP for a market facility is the ratio of the facility’s coefficient to that of the market fee; i.e., $w_{n}=\frac{c_{n}}{\lambda_{n}}$. This definition allows us to write the utility function in WTP space [34,35] as:

$$U_{njt}={-\lambda }_{n}P_{njt}+(\lambda_{n}w_{n})\prime x_{njt}+\zeta_{njt}$$ (5)

#### Attribute nonattendance

We also compared the heuristics respondents employed in the choice decision making process before and after the reminder. We are focusing on attribute nonattendance (ANA) as the most probable strategy that respondents employ to simplify their choice decision process in this context. ANA refers to the choice decision simplification strategy that decision makers employ through ignoring one or more facilities characterizing a market profile in the choice situation. ANA can be ‘stated’ whereby respondents indicate the attribute/attributes they ignored, or it can be ‘inferred’ based on the relative weights of the random coefficients of the utility model [21]. We did not generate data on stated nonattendance and, hence, we will be reporting inferred ANA. Clustering of respondents based on inferred ANA can only be done probabilistically [4].

We therefore estimated a series of latent class models (LCM) to map the ANA pattern among the sample respondents. In LCM setting, it is assumed that the respondents can be divided into a set of M classes or clusters of individuals distinguished by which of the attributes were considered in their choice process [4,38]. Within the context of the LCM, the logit probability function of an individual *n* belonging to a preference class *m* choosing an alternative from *J* alternatives can be specified as:

$$P(n,j|m)=\frac{exp(\beta_{m}^{\prime }x_{n,j})}{\Sigma_{j=1}^{J}exp(\beta_{m}^{\prime }x_{n,j})}$$ (6)

When the sorting of individuals is not observable, directly constructing the likelihood function for estimation of the parameters of the LCM model is not possible [4]. Therefore, the analyst needs to estimate a set of probabilities (ψ_*m*_) that each individual *n* falls into class *m*. Therefore, the marginal probabilities that individual *n* will choose alternative *j* is found by averaging over the classes, and given as:

$$P_{n,j}=\Sigma_{m=1}^{M}\psi_{m}\left(\frac{exp(\beta_{m}^{\prime }x_{n,j})}{\Sigma_{j=1}^{J}exp(\beta_{m}^{\prime }x_{n,j})}\right)\;where\;\Sigma_{m=1}^{M}\psi_{m}=1$$ (7)

In this case, the latent classes imply the clusters based on the patterns of attribute non-attendance *before* and *after* the nudge and relate to attendance in choice behavior (attendance heterogeneity) instead of groups of respondents with different intensities of taste heterogeneity. Therefore, number and type of facilities unattended to would decide whether a respondent belongs to a group or not.

Following the methodology suggested by [21,39] and [38], we estimated the LCMs gradually with the coefficients of the facilities assumed to be ignored set to zero. We did not consider all combinations [2^k^, where k is number of attributes] in analyzing the ANA. Only full attendance, full randomness [ignoring all], one-attributed nonattendance, and two-attribute nonattendance were considered.

### 3. Results and discussion

#### 3.1. The sample respondents

Our sample is composed of both men (73.65%) and women (26.35%). About 85% of the respondents were heads of their respective households. In fact, only 50.4% of the women respondents were heads of their households, whereas more than 97% of the male respondents headed theirs. The average respondent in our sample was 42.4 years old, had education of 4.3 years, had a family of 6 people, visited the livestock market about 7 times in a year walking for about 1.2 hours, and owned 1.12 hectares of land (Table 2). The mainstay of livelihood was reported to be farming by 96% of our respondents remotely followed by petty trading (2.29%) and running own small business other than farming (1.04%). Majority (96.35%) of the sample households owned livestock at the time of the survey. In tropical livestock units, the average small ruminant holding per household is only 0.92. This ownership ranges from none to 16.5 units.

**Table 2:** Summary of the characteristics of the sample respondents.

	N	Mean	St. Dv.	Min.	Max.	Kurtosis	Skewness
Age of respondent in years	960	42.4	12.65	10	87	2.82	.48
Education of the respondent in years	952	4.28	4.1	0	30	4.21	.89
Household size	960	5.88	2.22	1	17	3.39	.36
Walking distance to the nearest livestock market (hrs.)	960	1.22	.9	0	4	2.94	.76
Frequency of visit to livestock market in a year	956	6.49	10.83	0	120	27.69	4.2
Small ruminant owned—in TLU	960	.92	1.41	0	16.5	33.32	4.5
Farmland owned by the HH in hectare	952	1.12	.98	0	8.75	12.70	2.32

### 3.2. Comparison of the taste parameters and WTP values

Diagnostic tests were conducted to compare the taste parameters and the WTP values estimated on the datasets ‘*before’* and ‘*after*’ the reminder. The test parameters were compared based on conditional logit [CL] models (Table 3). We estimated the CL models and tested whether the two sets of taste parameters are equal using Swait and Louviere procedure [40]. The test checks whether the equality of the parameters implied by the conditional logit model estimated on the pooled data of the two DCE designs (Model 3) is acceptable. The test marginally rejected [p<0.1] the hypothesis that ‘*before’* and ‘*after*’ reminder observations have resulted in equal preferences for the market facilities. The scale parameter was found to be insignificant (Model 4) implying that the error variance was not explaining this difference between the taste parameters. This is an important observation in relation to the consistency of the choice behavior of the respondents. The reminder nudge has a considerable effect on the way they evaluate the market facilities and hence the choice of markets. The discussion in here needs to be seen in association with the choice simplification strategies discussed in section 3.4.

**Table 3:** Taste parameter estimates–CL model estimations.

	Model 1	Model 2	Model 3	Model 4
	(Before)	(After)	(Pooled)	(Pooled–HCL)
Opt out	-3.02^‡^	-3.28^‡^	-3.14^‡^	-3.10^‡^
	(0.09)	(0.09)	(0.06)	(0.08)
Fenced shed	0.40^‡^	0.44^‡^	0.42^‡^	0.42^‡^
	(0.03)	(0.03)	(0.02)	(0.02)
Unfenced shed	0.09^‡^	0.14^‡^	0.11^‡^	0.11^‡^
	(0.03)	(0.03)	(0.02)	(0.02)
Veterinary clinic	0.51^‡^	0.53^‡^	0.52^‡^	0.51^‡^
	(0.03)	(0.03)	(0.02)	(0.02)
Holding barn	0.27^‡^	0.36^‡^	0.31^‡^	0.31^‡^
	(0.03)	(0.03)	(0.02)	(0.02)
Watering trough	0.41^‡^	0.40^‡^	0.40^‡^	0.40^‡^
	(0.02)	(0.02)	(0.02)	(0.02)
Toilet with cleaner	0.35^‡^	0.38^‡^	0.37^‡^	0.36^‡^
	(0.03)	(0.03)	(0.02)	(0.02)
Toilet with no cleaner	0.24^‡^	0.16^‡^	0.20^‡^	0.20^‡^
	(0.03)	(0.03)	(0.02)	(0.02)
Feed shop	0.35^‡^	0.37^‡^	0.36^‡^	0.35^‡^
	(0.02)	(0.02)	(0.01)	(0.01)
Fenced shed	-0.07^‡^	-0.08^‡^	-0.08^‡^	-0.08^‡^
	(0.01)	(0.01)	(0.00)	(0.00)
Heteroscedasticity				
After = 1				0.03
Observations	17280	17280	34560	34560
LL	-4150.02	-4085.20	-8243.39	-8242.89
AIC	8320.04	8190.39	16506.78	16507.78
BIC	8397.61	8267.97	16591.28	16600.73

Note: Standard errors in parentheses.

* *p* < 0.10

^†^ *p* < 0.05

^‡^ *p* < 0.01. Model 4 is heteroscedastic conditional logit model with the reminder nudge being the only source of heterogeneity. LL stands for log likelihood, AIC for Akaike Information Criterion, and BIC is Bayesian Information Criterion.

The other test we conducted is to compare WTP values between *before* and *after* the information nudge. We started with mixed logit models estimated in preference space. In this section, we also assumed the price coefficient to be fixed to simplify the distributional challenges that arise in generating a random variable [WTP] as a ratio of two random variables [34,35]. The 95% confidence interval of the marginal WTP values (Table 4) was calculated using Krinsky-Robb method to ensure that percentiles are defined in case the moments of the WTP distributions are not defined [41].

**Table 4:** Comparing WTP values before and after attention reminder—Poe test results.

	Before reminder (WTP1)			After reminder (WTP2)			Poe test	
	mean	ll	ul	mean	ll	ul	Hypothesis	p value
Fenced shed	6.17	4.86	7.78	5.58	4.51	6.82	WTP1>WTP2	0.295
Unfenced shed	1.49	0.49	2.60	1.82	0.97	2.73	WTP1>WTP2	0.697
Veterinary clinic	7.55	6.20	9.44	6.45	5.40	7.77	WTP1>WTP2	0.137
Holding barn	4.24	3.17	5.74	4.38	3.46	5.63	WTP1>WTP2	0.583
Watering trough	5.95	4.87	7.60	4.85	4.05	5.95	WTP1>WTP2	0.091
Toilet with cleaner	5.04	3.89	6.59	4.54	3.60	5.72	WTP1>WTP2	0.283
Toilet with no cleaner	3.53	2.44	4.81	1.97	1.13	2.90	WTP1>WTP2	0.015
Feed shop	5.10	4.20	6.28	4.49	3.76	5.40	WTP1>WTP2	0.183

Note: WTP stands for willingness to pay. ll stands for lower limit and ul stands for upper limit of the confidence interval. The WTP values were generated based on mixed logit models estimated in preference space. The Poe tests for both mean and median differences were based on 1000 replications. The Stata command *mixlogit* [42] was used to estimate the mixed logit models and *mixlogitwtp* [43] was used to estimate the WTP values. The Poe test was conducted with a user written Stata command poetest written by Julian Sagebiel (www.slu.se/en/ew-cv/julian-sagebiel).

The Poe test revealed that most of the WTP values are not statistically different and only two facilities have; i.e., water troughs and toilets with no cleaner, statistically different values, albeit marginally, between *before* and *after* the reminder. The WTP values before the reminder for these two facilities were greater than the after-reminder values. We will discuss the WTP values in detail based on the estimations in WTP space in section 3.5.

In summary, the diagnostic tests have shown that the information nudge has affected the weights of the taste parameters and WTP for two of the facilities without considerably affecting the relative importance of the facilities.

### 3.3. Preference heterogeneity

Heterogeneity in mean preferences is an important result of stated choice analysis as it reveas the variations in interest among the sample population. Targeting of interventions benefits from information on heterogeneity and its sources. We have estimated different RPL models to compare the extent to which observed and unobserved heterogeneity is explained before and after the attention reminder. The first RPL model [not reported] included all interactions between the selected socioeconomic variables and the attributes in the choice experiment. Then, only significant interactions in either before or after-reminder RPL models were retained for the final estimation.

The model selection criteria show that the after-reminder data fit the heterogeneity in model slightly better. The results also show that the model coefficients are mostly heavier in the after-reminder model with slight trade off in efficiency (Table 5).

**Table 5:** Preference heterogeneity in mean *before* and *after* attention reminder.

	Model 1 [Before]		Model 2 [After]	
	Coeff.	St. error	Coeff.	St. error
***Non-random parameters in utility functions***				
Opt out indicator (1 = opted out)	3.852^‡^	0.129	4.354^‡^	0.151
***Random parameters in utility functions***				
Fenced market shed [SFEN]	.427^‡^	0.078	.441^‡^	0.087
Unfenced market shed [SUNF]	-0.103	0.128	.404^‡^	0.138
Veterinary clinic [VET]	.507^‡^	0.063	.648^‡^	0.071
Resting/holding shed [HLD]	.295^‡^	0.104	.576^‡^	0.117
Watering trough [WAT]	.494^‡^	0.030	.528^‡^	0.033
Toilet with a cleaner [TCLN]	.347^‡^	0.048	.489^‡^	0.054
Toilet with no cleaner [TNCL]	.492^‡^	0.117	-0.017	0.128
Feed stall/shop [FDSH]	.409^‡^	0.030	.484^‡^	0.034
Market fee [FEE]	-.049*	0.028	-0.044	0.031
***Heterogeneity in mean coefficients***				
SFEN: Gender [1 = male]	.141*	0.082	.176*	0.093
SFEN: small ruminant wealth in TLU	-0.014	0.029	.061*	0.033
SUNF: Age in years	.006^†^	0.003	-0.003	0.003
SUNF: Small ruminant wealth in TLU	0.001	0.030	-.084^‡^	0.032
SUNF: Wag area [cf. Horro]	.163^‡^	0.043	.130^‡^	0.047
VET: Gender [1 = male]	.177^†^	0.069	0.081	0.076
VET: Wag area [cf. Horro]	.136^‡^	0.039	.144^‡^	0.043
HLD: Gender [1 = male]	.234^‡^	0.061	.125*	0.068
HLD: Age in years	0.001	0.002	-.008^‡^	0.002
HLD: Household size	-.024^†^	0.011	.026^†^	0.012
WAT: Menz area [cf. Horro]	0.022	0.027	-.058^†^	0.029
TCLN: Small ruminant wealth in TLU	.062^‡^	0.023	0.015	0.024
TNCL: Age in years	-.004*	0.003	.005*	0.003
FDSH: Small ruminant wealth in TLU	.035^†^	0.016	0.023	0.018
FDSH: Menz area [cf. Horro]	-.054*	0.028	-.112^‡^	0.033
FDSH: Wag area [cf. Horro]	0.013	0.033	.095^†^	0.038
FEE: Age in years	-0.001	0.001	-.002^†^	0.001
FEE:Menz area [cf. Horro]	-.054^‡^	0.009	-.063^‡^	0.010
***Standard deviations of the random parameters***				
Fenced market shed (n)	.413^‡^	0.072	.606^‡^	0.068
Unfenced market shed (n)	.305^‡^	0.084	.266^†^	0.110
Veterinary clinic (n)	.333^‡^	0.061	.409^‡^	0.064
Resting/holding shed (n)	0.113	0.110	.198^†^	0.079
Watering trough (n)	0.111	0.093	.152*	0.088
Toilet with a cleaner (n)	0.187	0.116	.222^†^	0.111
Toilet with no cleaner (n)	.256^‡^	0.080	.350^‡^	0.081
Feed stall/shop (n)	.205^‡^	0.047	.320^‡^	0.041
Market fee (n)	.153^‡^	0.010	.171^‡^	0.011
N	5760		5760	
LL	-3986.164		-3888.957	
AIC/N	1.397		1.363	
McFadden R^2^	0.37		0.385	

Note

^‡^, ^†^, and * denote p<0.01, p<0.05 and p<0.1. N stands sample size, LL stands for log likelihood at convergence. AIC denotes Akaike Information Criterion. The models were estimated using NLOGIT 6. The estimation commands and additional results are available upon request from the corresponding author.

The reminder does not seem to have a considerable effect on the relationship between gender of the respondent, and fenced market shed and holding barns. Compared to females, male respondents have higher interest in fenced sheds and holding barns both before and after the reminder. Similarly, regardless of the reminder, respondents in Wag area, compared to those in Horro, are more interested in unfenced market sheds and veterinary clinics around the markets. The significant disinterest of respondents in Menz area, compared to those in Horro, in feed shops and market fees was not affected by the attention reminder.

There are some variations in the heterogeneity in mean coefficients of the two models. For instance, before the reminder, small ruminant ownership in TLU explained heterogeneity around mean preferences for toilets with no cleaners and feed shops in the markets. After the reminder, small ruminant wealth was positively related to interest in fenced sheds and negatively related to interest in unfenced market sheds (Table 5). Another difference is the importance of age of the respondents in explaining heterogeneity around the mean preferences after the reminder. Age was observed to have a more pronounced and negative relationship with interest in holding barns and market fee.

The models have also resulted in opposite results in explaining heterogeneity. Before the reminder, household size is negatively related to interest in holding barn before the reminder and positively related after the reminder. Similarly, age of the respondent is negatively related to interest in toilets with no cleaners before the reminder and the relationship turns positive after the reminder. Both directions of the relationship can be justified given the characteristics of the farm households. Yet, the after-reminder estimates seem to be more plausible. For instance, given the multiple purposes of visiting markets in rural Ethiopia, farm households with large family size might need to accomplish many more activities in the markets and therefore might be more interested in a safe place to keep their animals. Once again, as age increases sense of responsibility develops. Therefore, it is safe to assume that older respondents see the virtue of having a toilet even without a cleaner in the markets. Generally, we believe that the heterogeneity in mean preferences is better explained by the data generated after the reminder.

### 3.4. Attribute nonattendance

In this section we present the attribute non-attendance (ANA) *before* and *after* the information nudge. Table 6 presents four Latent Class Model (LCM) models that quantified the probability of individual respondents falling into classes of different ANA levels. The first two models [Model 1(b) and Model 2(b)] were estimated on the data *before* the reminder and the other two models [Model 1(f) and Model 2(f)] on the data *after* the reminder.

**Table 6:** Probabilities of ANA classes before and after the nudge–LCM results.

ANA Class		Model 1(b)	Model 2(b)	Model 1(f)	Model 2(f)
		Cl. prob. (%)	Cl. prob. (5%)	Cl. prob. (%)	Cl. prob. (%)
1	Full attendance (no ANA)	0.61	6.32	0.45	18.04
2	No attendance (random choice)	0.37	0.32	4.68	0.72
3	Fenced shed only	12.57		19.40	
4	Unfenced shed only	0.70		0.39	
5	Veterinary clinic	2.19		10.57	
6	Holding shed	0.26		0.28	
7	Watering trough	3.61		0.43	
8	Toilet with cleaner	0.63		3.51	
9	Toilet with no cleaner	0.38		0.35	
10	Feed shop	1.71		11.21	
11	Market fee	76.96		48.73	
12	Fenced shed and vet clinic				9.77
13	Fenced shed and water trough		10.23		
14	Fenced shed and market fee		32.49		26.08
15	Unfenced shed and market fee		12.83		
16	Vet clinic and market fee		14.29		20.36
17	Holding barn and market fee				8.48
18	Watering trough and market fee		9.78		
19	Feed shop and market fee		13.75		16.55

Note: Cl. prob. stands for class probability and indicates size. Model 1(b) and Model 2(b) are estimations on the before reminder data. Model 1(f) and Model 2(f) are estimations on the after reminder data. The LCM models were estimated using LatentGOLD 5.1.

Model 1(b) and Model 1(f) are the first batch of estimations with eleven ANA classes. Class 1 represents the conventional compensatory substitution of attributes specification (full attendance, fully compensatory class), and class 2 represents the decision rule with the assumption that all services were ignored (total random choice). Class 3 to class 11 are specifications with one facility non-attendance each.

The second set (not reported) includes Class 1 and Class 2 estimated above, one-facility non-attendance classes with class size larger than 5%, and non-attendance of all combinations of two facilities. The third set includes the first two classes again and all one-facility and two-facility non-attendance classes with size larger than 5%. For the data before the reminder, LCM with eight classes was estimated [Model 2(b)]. For the data after the reminder, LCM with seven classes was estimated [Model 2(f)]. The parameter estimates are constrained to be equal across classes to control for other sources of preference heterogeneity among individuals other than the probabilistic decision rule or heuristics they employ [21].

Model 1(b) and Model 1(f) show that three fourth of the respondents ignored the market fee before-reminder whereas only about half the respondents ignored it after-reminder. The reminder seems to have distributed the attention over all facilities as expected. Model 2(b) and Model 2(f) show that full attendance increased quite significantly after the reminder from 0.61% to 18.04%. The number of ANA classes is less in the after-reminder case. However, the size of the classes in the *after*-reminder estimation is larger in two of the three common classes. There are three ANA classes unique for the *before*-reminder estimation, whereas there are only two classes unique to the *after*-reminder case.

Generally, the reminder increased probability of full attendance, and concentrated non-attendance on fewer classes. In both cases, the two largest ANA classes are fenced shed & market fee, and veterinary clinic & market fee [Model 2(b) and Model 2(f)]. The payment mechanism [market fee] is the main denominator in the two facility ANA classes. The payment mechanism in DCEs is usually the one subjected to non-attendance [44,45]. It is clear that the fee is the source of disutility for the respondents and probably the most hypothetical component of many of the DCEs. It is not, therefore, unexpected that our respondents paid less attention to the fee attribute compared to the services.

The effects of the information nudge on ANA reveal that respondents did not comply with the axiom of fully compensatory continuous preferences and a simple reminder seems to make the respondents decide more in line with the rationality assumptions of consumer behavior theory. It is clear that respondents have either put more effort in the choice process or were forced to change the relative importance they attach to the different services after the nudge.

### 3.5. Attention reminder and marginal willingness to pay (mWTP) estimates

In this section, we report results of estimations of the MXL model [46,47] estimated in WTP space (Table 7). The main intention here is to the see the effect of the *nudge* on the marginal WTP values of the market services.

**Table 7:** Willingness to pay for market facilities.

	Model 1 (Before)		Model 2 (After)		Model 3 (Pooled)	
Mean	Coeff.	St.err.	Coeff.	St.err.	Coeff.	St.err.
Opt out	-60.174^‡^	6.046	-49.105^‡^	4.201	-57.970^‡^	3.915
Fenced market shed	6.052^‡^	0.736	5.491^‡^	0.596	5.483^‡^	0.517
Unfenced market shed	1.543^‡^	0.512	1.914^‡^	0.425	1.738^‡^	0.333
Veterinary clinic	7.502^‡^	0.819	6.421^‡^	0.625	6.902^‡^	0.544
Resting/holding shed	4.219^‡^	0.628	4.416^‡^	0.538	3.936^‡^	0.438
Watering trough	5.891^‡^	0.654	4.807^‡^	0.478	5.369^‡^	0.426
Toilet with a cleaner	4.990^‡^	0.649	4.473^‡^	0.513	4.677^‡^	0.401
Toilet with no cleaner	3.442^‡^	0.576	2.009^‡^	0.457	2.860^‡^	0.414
Feed stall/shop	5.072^‡^	0.530	4.443^‡^	0.417	4.559^‡^	0.351
Market fee (*-1)	-2.535^‡^	0.105	-2.286^‡^	0.089	-2.392^‡^	0.067
Fenced shed*after					0.601	0.577
Vet clinic*after					0.156	0.500
Holding barn *after					0.847*	0.460
Watering trough*after					-0.096	0.385
Toilet no cleaner*after					-0.329	0.483
Feed shop*after					0.379	0.347
Standard dev. (Heterogeneity in mean coefficients)						
Opt out	26.782^‡^	3.540	-20.444^‡^	2.484	25.981^‡^	2.375
Fenced market shed	4.648^‡^	1.024	5.564^‡^	0.777	4.022^‡^	0.554
Unfenced market shed	3.583^‡^	1.116	1.958	1.428	3.106^‡^	0.511
Veterinary clinic	3.480^‡^	0.886	-4.041^‡^	0.641	-3.085^‡^	0.460
Resting/holding shed	2.041^†^	0.894	2.107^‡^	0.652	1.117	0.693
Watering trough	-0.257	2.075	1.319	0.930	1.183^†^	0.561
Toilet with a cleaner	-2.406*	1.249	2.153^†^	0.966	-2.207^‡^	0.564
Toilet with no cleaner	2.514*	1.285	3.403^‡^	0.710	2.581^‡^	0.604
Feed stall/shop	2.444^‡^	0.580	2.788^‡^	0.431	2.274^‡^	0.298
Market fee (*-1)	0.242*	0.133	-0.027	0.129	0.301^‡^	0.059
Fenced shed*after					4.267^‡^	1.028
Vet clinic*after					3.324^‡^	0.805
Holding barn *after					-2.077^‡^	0.766
Watering trough*after					0.657	1.618
Toilet no cleaner*after					2.706^‡^	1.039
Feed shop*after					1.736^†^	0.701
*N*	17280		17280		34560	
LL	-4031.180		-3946.745		-7823.738	
AIC	8102.361		7933.490		15711.476	
BIC	8257.507		8088.636		15981.891	

Note

* *p* < 0.10

^†^ *p* < 0.05

^‡^ *p* < 0.01. N is number of observations. LL stands for log likelihood, AIC for Akaike Information Criterion, and BIC is Bayesian Information Criterion.

Model 1 is estimated on the before-reminder DCE dataset. Model 2 is on the after-reminder dataset. We estimated a fully saturated model (not reported for brevity reasons) with the effect of the attention reminder captured as interaction of reminder dummy and the facilities. Model 3 is a more parsimonious saturated model with only interactions which showed significant heterogeneity around the mean marginal WTP were included.

A naked eye comparison of the *before* [Model 1] and *after* [Model 2] reminder models shows that the mWTP is generally slightly smaller after the reminder. Only two of the least favored facilities; i.e., unfenced shed and holding barn, gained some weight in the *after*-reminder estimation. In fact, the goodness of fit indicators favour the after-reminder model indicating that the behavior described by the models is more in line with the behavior respondents showed after they were reminded of full attention. The saturated model (Model 3), however, fits better to the pooled dataset given the number of observations. Our discussion will focus on Model 4.

Referring to Model 3, the farm households are willing to pay 7.073 birr [~US 0.26 cents] per sheep or goat for a veterinary service in the livestock market (Model 3). This is 4, 2.6, 1.6, 1.5, 1.3 and 1.2 times the mWTP for unfenced shed, toilet with no cleaner, holding barn, toilet with cleaner and feed shop, watering trough, and fenced shed. In all estimations, veterinary clinic, fenced shed, and watering trough are the three most important facilities for the sample farm households.

Only the mean of the marginal WTP (mWTP) for holding barn was affected by the attention reminder (Models 3). However, the heterogeneity around the mean mWTP of five facilities were significantly affected by the reminder [Model 3]. The relative interest in toilets with no cleaners significantly decreased after the reminder, whereas the sensitivity significantly increased for fenced sheds, veterinary clinics, watering troughs, and feed shops.

Heterogeneity around the mean mWTP is more pronounced before the reminder. This is an indication that the reminder has reduced the stochasticity of the choice behavior. This is in line with the observations reported by [48] who studied the effect of response time on error variance in DCE surveys and [49] who studied whether a trap question affects the variance of the WTP values.

## 4. Conclusion

Development of the livestock markets is an investment that Ethiopia needs to make as part of the effort to transform agrarian livelihoods. This investment needs to be evidence-based to ensure that the limited resources of the country are used efficiently. The evidence presented here contributes towards the national agricultural growth and transformation agenda. The rural communities studied are willing to pay for the livestock market facilities.

The analyses done consistently showed that veterinary clinic, fenced market shed, and watering troughs, in order, to be the three most important market facilities from farm households’ perspective. Prioritization of the investment that needs to be made to develop livestock markets and determination of the service charges need to take these preferences and WTP values into consideration.

These preferences were found to be consistent even after the introduction of a simple information nudge meant to influence the heuristics the respondents apply in the decision making process. The nudge in our DCE resulted in some analytically interesting observations. The taste parameters and WTP values based on preference space estimations showed marginal difference between *before* and *after* the information nudge. WTP values were slightly smaller after the reminder. Preference heterogeneity around average taste parameters was more pronounced after the reminder. The reminder increased fully compensatory behavior which an important aspect of rational consumption behavior.

The reminder also reduced the likelihood of ignoring the cost attribute [market fee]. The fact that respondents notice that they do not have to pay the specified amount could be the reason why they ignore it in the first place. When nudged to consider all attributes, they might, therefore, look into the fee attribute more seriously than otherwise implying increased compensatory decision-making behavior. The reminder nudge generally increased full compensatory behavior and evened out attribute non-attendance in the decision making process.

We believe that this paper addresses an interesting question with regard to the effect of a simple reminder nudge on attention and willingness to pay for attributes in a discrete choice experiment setup. However, there are a number of interesting questions that need to be addressed through a more focused research. First, empirical evidence needs to be generated on how nudges shall be designed and introduced in discrete choice experiments. We are not aware of any research in this line and the limited effort to address the decision making process in DCEs focused only on reducing hypothetical bias through cheap talk, e.g., [50] and [51]. Second, what are the implications of the manipulability of heuristics in stated choices for modeling and development/policy decision making? The limited effort in describing the level and causes of cognitive strategies to simplify choice decisions needs to be enhanced and evolve to identifying strategies to manipulate cognitive strategies of individuals.

There are a couple of glaring limitations in our study that might also translate to interesting questions among researchers in the field. First, our study does not look into the relationship between the nudge and hypothetical bias. The market facilities studied are not available to farming communities yet, and we could not find any reliable references to assess the potential impact of the nudge on hypothetical bias. We encourage further investigation of the relationship between such nudges and hypothetical biases in choice experiments. Second, we did not include pastoral and agro-pastoral areas in our study. Although the livestock population is not as enormous as it is in the midlands and highlands of the country, the livelihoods of pastoralists and agro-pastoralists depend almost entirely on their livestock. Therefore, these market facilities could be more rewarding in terms of improving rural wellbeing in these environments. There might also be an important contrast between pastoralist and sedentary farmers in their reactions to reminder nudges.
